# Compulsive methamphetamine self-administration in the presence of adverse consequences is associated with increased hippocampal mRNA expression of cellular adhesion molecules

**DOI:** 10.3389/fnmol.2022.1104657

**Published:** 2023-01-13

**Authors:** Ceiveon Munoz, Subramaniam Jayanthi, Bruce Ladenheim, Jean Lud Cadet

**Affiliations:** Molecular Neuropsychiatry Research Branch, DHHS/NIH/NIDA, Intramural Research Program, Baltimore, MD, United States

**Keywords:** methamphetamine, hippocampus, gene expression, electric foot-shocks, cell adhesion

## Abstract

Methamphetamine (METH) is a popular but harmful psychostimulant. METH use disorder (MUD) is characterized by compulsive and continued use despite adverse life consequences. METH users experience impairments in learning and memory functions that are thought to be secondary to METH-induced abnormalities in the hippocampus. Recent studies have reported that about 50% of METH users develop MUD, suggesting that there may be differential molecular effects of METH between the brains of individuals who met criteria for addiction and those who did not after being exposed to the drug. The present study aimed at identifying potential transcriptional differences between compulsive and non-compulsive METH self-administering male rats by measuring global gene expression changes in the hippocampus using RNA sequencing. Herein, we used a model of METH self-administration (SA) accompanied by contingent foot-shock punishment. This approach led to the separation of animals into shock-resistant rats (compulsive) that continued to take METH and shock-sensitive rats (non-compulsive) that suppressed their METH intake in the presence of punished METH taking. Rats were euthanized 2 h after the last METH SA plus foot-shock session. Their hippocampi were immediately removed, frozen, and used later for RNA sequencing and qRT-PCR analyses. RNA sequencing analyses revealed differential expression of mRNAs encoding cell adhesion molecules (CAMs) between the two rat phenotypes. qRT-PCR analyses showed significant higher levels of *Cdh1*, *Glycam1*, *and Mpzl2* mRNAs in the compulsive rats in comparison to non-compulsive rats. The present results implicate altered CAM expression in the hippocampus in the behavioral manifestations of continuous compulsive METH taking in the presence of adverse consequences. Our results raise the novel possibility that altered CAM expression might play a role in compulsive METH taking and the cognitive impairments observed in MUD patients.

## Introduction

Methamphetamine (METH) is an amphetamine-type psychostimulant drug which is among the most misused substances in the world (UNODC (United Nations Office on Drug and Crime), 2022). METH can be taken orally, *via* snorting, smoking, and intravenously, with the intravenous route being mostly involved in METH-related overdose deaths (Han et al., 2021; Jones et al., 2022). Humans who use the drug experience a multitude of physiological, neurological, and behavioral sequelae including neuroinflammatory responses and cognitive impairments (Moszczynska and Callan, 2017; Paulus and Stewart, 2020). METH exerts its effects *via* the release of monoamines such as dopamine and noradrenaline from synaptic vesicles and causing changes in their metabolism (Moszczynska and Callan, 2017; Jayanthi et al., 2021; Daiwile et al., 2022b).

About 50% of METH users develop METH use disorder (MUD) which is characterized by repeated drug misuse, loss of control over drug use, compulsive use despite negative consequences, and multiple relapse episodes, according to the Diagnostic Statistical Manual (DSM5) of the American Psychiatric Association (APA) DSM5 (2022). Patients also show changes in learning and memory functions (Shukla and Vincent, 2021) that are subserved by the hippocampus (Golsorkhdan et al., 2020). The substrates for METH-associated cognitive disturbances might include epigenetic modifications, altered gene expression, changes in synaptic plasticity, and dysfunctional neurotransmission (Chojnacki et al., 2020; Golsorkhdan et al., 2020; Shukla and Vincent, 2021). This line of reasoning suggests that METH might influence the expression of genes including cell adhesion molecules (CAMs) that participate in regulating hippocampal synaptic plasticity (Cotman et al., 1998; Gnanapavan and Giovannoni, 2013) and other drug-induced neuroadaptions during addictive processes (Muskiewicz et al., 2018).

As a step toward identifying genes that might be differentially regulated during compulsive METH taking in the presence of adverse consequences, we used the discovery approach of RNA sequencing using the model of foot-shock-induced compulsive and non-compulsive rat METH SA (Cadet et al., 2016, 2019; Subu et al., 2020; Jayanthi et al., 2022). We also used quantitative PCR to validate changes in some CAMs that were identified by RNA Sequencing as showing differential expression between compulsive and non-compulsive METH takers in the presence of foot-shocks. Herein, we discuss the potential role of hippocampal CAMs in mediating METH-induced compulsive METH taking.

## Materials and methods

### Animals and drug treatment

Male Sprague–Dawley rats each weighing 350-400 g were used (Charles River Labs, Raleigh, NC, United States). Animals were housed in a humidity and temperature controlled (22.2 ± 0.2°C) room with free access to food and water. All animals were handled as outlined in the Guide for the Care and Use of Laboratory Animals (ISBN: 0–309–05377-3) and animal protocol was approved by the National Institute of Drug Abuse Animal Care and Use Committee (NIDA, ACUC).

### Intravenous surgery

Rats were anesthetized using ketamine (100 mg/kg i.p.) and xylazine (5 mg/kg i.p.) and silastic catheters were put into the jugular vein. After surgery, animal health was monitored daily, and catheters were flushed with sterile saline containing gentamicin (Butler Schein; 5 mg/ml) and allowed to recover for 5–10 days before start of METH self-administration training. Upon wake from anesthesia, rats received meloxicam (1 mg/kg s.c.), as an analgesic, and a second dose the following day.

### Training and punishment phases

As mentioned, our METH self-administration training procedure was performed according to the previously designed protocol for our lab (Cadet et al., 2016; Subu et al., 2020; Jayanthi et al., 2022). Rats were housed in self-administration chambers with free access to food and water, made available in water bottles and feeders hanging from each chamber wall. We trained rats to self-administer dl-methamphetamine HCl (NIDA), by pressing an active infusion pump lever, for three 3-h sessions per day, for 21 days. To achieve this, rat catheters were connected to a modified cannula (Plastics One, Minneapolis, MN) attached to a liquid swivel (Instech Laboratories, Inc., Plymouth Meeting, PA, United States) *via* polyethylene-50 tubing protected by a metal spring. To prevent overdose, each 3-h self-administration training session was separated by a 30-min timeout during which the animals had no access to the active lever. Rats self-administered METH at a dose of 0.1 mg/kg/infusion over 3.5 s, and the number of infusions was limited to 35 per each 3-h training session, and each pressed lever infusion was given a 20-s recess timeout. To reinforce, active lever presses were accompanied by a compound tone-light cue, and presses on inactive lever induced no reinforcement cues. Rats were trained 5 days a week, with 2 days off to minimize weight loss for a total of 21 days of METH self-administration training. During the 2 days off, rats remained housed in chambers but were disconnected from the intravenous self-administration connection and cannulas were covered with dust prevention caps. We started self-administration sessions at the onset of the dark cycle and sessions began with insertion of the active lever and the illumination of a red light that remained on during the entire 3 h session. At the end of each session, and during 30-min timeouts, the red light was turned off, and the active lever was removed. Control rats self-administered saline, under the same conditions.

During the 8-day foot-shock punishment phase, rats continued METH self-administration for 8 days straight under the same 3-h session schedule used during the training phase. However, a 0.5-s foot-shock was delivered through the grid floor on 50% of all active METH lever presses. This electrical current was set at 0.18 mA on day 1, which was increased as the 8-day foot-shock phase proceeded. Shock intensity was set to 0.24 mA on day 2, 0.30 mA on days 3–5, and 0.36 mA on days 6–8. As an important addition, some control rats that self-administered saline were “yoked” connected to a METH taking rat and received a contingent shock whenever the rat it was paired to pressed for METH. As a control for the effects of shock on biochemical and molecular markers within the brain, the METH taking rat and the yoke-saline rat that it was paired to, received the same number of foot-shocks. Totally, after the 8-day foot-shock phase was complete, rats were separated into 5 groups CT (Saline, no foot-shock), SR (Methamphetamine, Shock Resistant), SS (Methamphetamine, Shock Sensitive), YSR (Saline, SR contingent foot-shock), and YSS (Saline, SS contingent foot-shock).

### RNA extraction and sequencing

Immediately after the last 3 h foot-shock session, rats were euthanized by decapitation, and the brains were removed and dissected by region. We dissected out the entire hippocampus using the coordinates A/P -5 to -7 mm bregma, mediolateral ±6 mm, D/V -2 to -8 mm, corresponding to *The Rat Brain in Stereotaxic Coordinates* (Paxinos and Watson, 2013).Total RNA was extracted from samples of the total hippocampal region using Qiagen RNeasy Mini kit (Qiagen, Valencia, CA, United States). RNA integrity was assessed using an Agilent 2,100 Bioanalyzer (Agilent, Palo Alto, CA, United States), and RNA samples showed no signs of degradation. RNA sequencing was performed by GeneWiz (GeneWiz, South Plainfield, NJ, United States) using Illumina HiSeq instrument according to manufacturer’s instructions and was converted into fastq files and de-multiplexed using the Illumina bcl2fastq 2.17 software. The samples were sequenced using a 2×150bp Paired End (PE) configuration (GeneWiz, South Plainfield, NJ, United States). Sequence reads were filtered to remove any poor-quality reads using Trimmomatic v.0.36 and this data has been submitted to Gene Expression Omnibus^1^ under accession number GSE203268.

### Ingenuity pathway analysis

We used Ingenuity Pathway Analysis (IPA) software (Qiagen, Valencia, CA, United States) to analyze genes that showed differential expression in RNA sequencing data and identify molecular functions, biochemical networks, and validated canonical gene pathways.

### Quantitative RT-PCR analysis of mRNA

To analyze mRNA levels, for each sample in each group (CT, SR, SS, YSR, YSS), we reversed-transcribed individual total RNA into cDNA using Advantage RT-for-PCR kit (Clontech, Mountain View, CA, United States). Using this kit, 500 nanograms(ng) of RNA was reversed-transcribed with oligo dT primers to create cDNA. We generated PCR primers using NIH accredited NCBI primer-BLAST website and ordered gene-specific primers from the Synthesis and Sequencing Facility of Johns Hopkins University (Baltimore, MD, United States). RT-qPCR was performed with the Roche LightCycler 480 II using iQ SYBR Green Supermix (Bio-Rad Laboratories, Hercules CA, United States). Relative amounts of mRNA in each sample were normalized to a combined mean of OAZ1 and Clathrin mRNA, used as housekeeping reference genes.

### Statistical analysis

Behavioral data, RNA sequencing data, and qRT-PCR data were analyzed using the statistical program GraphPad Prism version 9 (Dotmatics, San Diego, CA, United States). Behavioral data was analyzed using repeated measures two-way ANOVA. For the behavioral experiments, the dependent variable used was the number of METH or saline infusions during the 21-day training and 8-day foot-shock phases, for each group. RNA sequencing and qRT-PCR data was analyzed using one-way ANOVA followed by Tukey’s multiple comparison *post hoc* test. Statistical significance level for all tests was set to 0.05, and the null hypothesis was rejected at value of *p*<0.05.

## Results

### Foot-shocks separate methamphetamine self-administering rats into compulsive (shock-resistant) and non-compulsive (shock-sensitive) behavioral phenotypes

**Figure 1:**
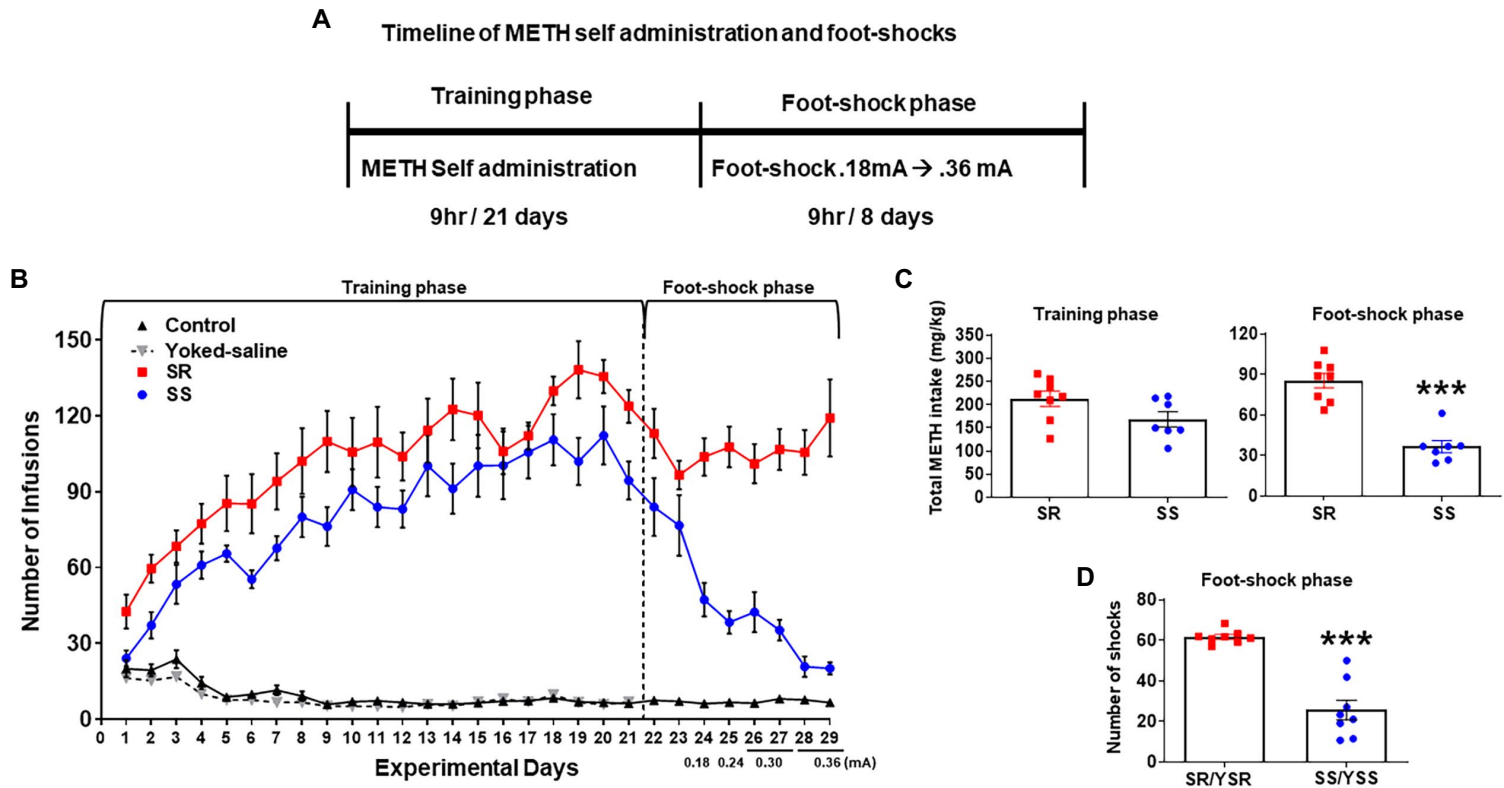
Contingent foot-shocks during METH self-administration (SA) are associated with continued compulsive drug taking behaviors. **(A)** Experimental timeline for METH SA training and punishment phases. **(B)** Average number of infusions (METH or saline) over the training phase (21 days) showed increases in METH infusions during SA training. The introduction of contingent shocks for 8 days suppressed lever pressing in non-compulsive, shock-sensitive (SS, *n* = 7) rats but not in shock-resistant rats (SR, *n* = 8). **(C)** Average total METH intake of SR rats (212.6 ± 15.35 mg/kg) and SS rats (168.1 ± 14.99 mg/kg) during the training phase (*first panel*) showed no significant differences. Foot-shocks (*second panel*) had significant differences in METH intake between SR rats (85.4 ± 5.38 mg/kg) and SS rats (36.5 ± 4.21 mg/kg). **(D)** Total numbers of shocks administered to the SR rats and their yoked-shock controls (YSR) were significantly higher than those received by SS rats and their yoked-shock control group (YSS). *Key to statistics:* ***p < 0.001 significant SR vs. SS.

Figure 1A shows the experimental timeline of the behavioral experiment. It includes METH SA training and contingent foot-shock phases. Figure 1B depicts the number of infusions of either METH or saline during the 21-day SA training period. All METH-trained rats (*n* = 15) significantly increased their drug intake whereas control rats (*n* = 9) did not change their saline intake during the training phase (Figure 1B). The repeated measure analysis using 2-way ANOVA for the SA training phase included the between-subject factor of groups: control (CT *n* = 9), yoked saline (YS *n* = 19), shock-resistant (SR *n* = 8), and shock-sensitive (SS *n* = 7), and the within-subject factor of training days (experimental days 1–21). There were significant differences in the number of infusions between groups [*F*_(3_,_35)_ = 139.9, *p* < 0.0001] over training days [*F*_(20_,_700)_ = 34.29, *p* < 0.0001], with there being significant interaction of day × group [*F*_(60_,_700)_ = 21.31, *p* < 0.0001].

On day 22, foot-shocks were introduced and increased in intensity from 0.18–0.36 mA over 8 days (experimental days 22–29). Foot-shocks helped to separate the rats into 2 different behavioral phenotypes. The group of rats that continued to compulsively press the active lever for METH infusions despite foot-shocks were labeled shock-resistant (SR) or compulsive METH takers (*n* = 8) whereas the rats that decreased their active lever pressing under punishment were termed shock-sensitive (SS) or non-compulsives (*n* = 7; Figure 1B). The repeated measures analysis for the foot-shock phase included the SR and SS groups, and the within-subject factor of days (experimental days 22–29). We found significant differences in the number of METH infusions between groups [*F*_(1,13)_ = 46.53, *p* < 0.0001] and over shock days [*F*_(7,91)_ = 6.654, p < 0.0001]. The interaction of day × group [F_(7,91)_ = 9.348, p < 0.0001] was also significant.

The total METH intake (mg/kg) of the SR and SS rats during the training and foot-shock phases are shown in Figure 1C. During SA training, all METH SA rats took comparable amounts of the drug. During the foot-shock/punishment phase, both groups decreased their drug intake, with the SS rats decreasing their active lever pressing significantly more than the SR rats. Overall, SS rats took significantly less METH [*F*_(1,6)_ = 66.86; *p* = 0.0002; Figure 1C] and received significantly less foot-shocks [F_(1,13)_ = 64.69; *p* < 0.0001; Figure 1D] than SR rats. The 2-way ANOVA for number of foot-shocks was also significant for groups [F_(1,13)_ = 64.69, p < 0.0001], days [F_(7,91)_ = 6.920, *p* < 0.0001], and interaction of day × group [F_(7,91)_ = 9.892, p < 0.0001; Figure 1D].

As mentioned in the Methods, some rats that self-administered saline were individually connected to METH taking rats. These yoked rats received foot-shocks whenever the METH SA rat to which they were paired received a shock, such that individual yoked-saline rats and paired METH-taking rats received the same number of shocks by the end of the behavioral experiment. These paired rats were labeled yoked shock-resistant (YSR) or yoked shock-sensitive (YSS).

### Genome-wide transcriptional analyses revealed significant differences in gene expression between hippocampus of compulsive vs. non-compulsive methamphetamine taking rats

To identify potential transcriptional changes in the resistant and sensitive rats, we used RNA sequencing using RNA obtained from whole hippocampal tissues (CT, *n* = 7; SR, *n* = 7; SS, *n* = 6; YSR, *n* = 5; YSS, *n* = 5). We then calculated RNA expression fold changes to find differentially expressed genes (DEGs) between 8 pairings (SR vs. CT, SS vs. CT, YSR vs. CT, YSS vs. CT, SR vs. SS, SR vs. YSR, SS vs. YSS, YSR vs. YSS).

**Figure 2:**
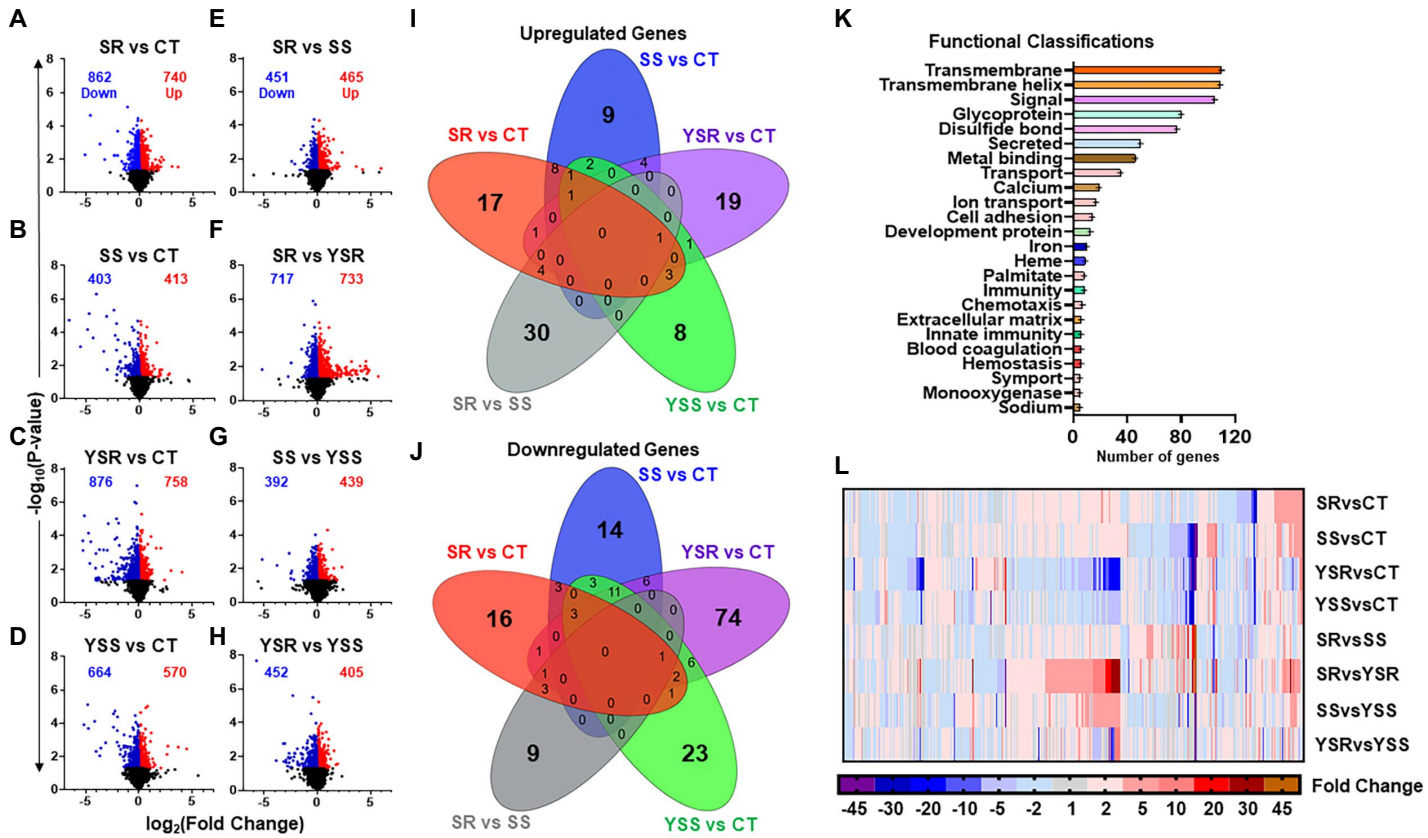
**(A–H)** Volcano plots illustrating the number of significant genes (*p* < 0.05) between each pair-wise comparison. Volcano plots were created using GraphPad Prism version 9 (Dotmatics, San Diego, CA) with scaled fold changes [log_2_ (foldchange)] on the *X*-axis, and value of ps [−log_10_ (value of *p*)] scaled on *Y*-axis. **(I)** The Venn diagram shows significant upregulated genes (*p* < 0.05, fold change 1.7F) in control comparisons, with unique genes specific to that comparison in outer rings. This Venn analysis identified 30 upregulated genes that were found exclusively in the SR vs. SS comparison. **(J)** Overlap of downregulated genes showed fewer total genes in the SR vs. SS comparison. There was also a higher number of 354 downregulated genes in the SS vs. CT comparison. **(K)** Functional annotation analysis of differentially expressed genes (DEGs), using DAVID bioinformatics database, revealed their functional classifications. Prominent among these annotations were transmembrane function, cell signaling, ion transport, and cell adhesion molecules. **(L)** Heatmap illustrating fold changes between pair-wise comparisons of DEGs that met the criterion of 1.7-fold-change and *p* < 0.05, with blue to purple indicating downregulated gene expression, and red to orange indicating increased gene expression.

Using GraphPad Prism (Version 9), we created volcano plots to visualize the entire sets of DEGs obtained from the sequencing data (Figures 2A,H). The volcano plots, showing up- and downregulated genes, were plotted using fold-changes scaled on the x-axis, and value of p shown on the y-axis. Note that SR group comparisons (SR vs. CT and SR vs. YSR; Figures 2A,F) showed more total DEGs than the SS group comparisons (SS vs. CT and SS vs. YSS; Figures 2B,G). The YSR vs. CT (Figure 2C) and YSS vs. CT (Figure 2D) comparisons showed clearly that shock-induced stress had significant effects on hippocampal gene expression.

To identify potential overlaps of DEGs between the various comparisons, we created Venn diagrams^2^ (Figures 2I,J). For these comparisons, we used fold-changes of greater or less that 1.7-fold and *p*-values of 0.05. We found that 30 upregulated genes (Figure 2I) and 9 downregulated genes (Figure 2J) were unique to the SR vs. SS comparison. Figures 2I,J showed very little overlaps between the pairwise comparisons.

We used DAVID gene functional annotation tool (DAVID Bioinformatics) to examine functional classifications for the significant DEGs among the eight comparisons (Figure 2K). The DEGs fall within functional classes that included cell signaling, metal-binding, transport of ions, calcium signaling, and cell adhesion (Figure 2K). Figure 2L shows a heatmap (GraphPad Prism) that illustrates relative changes in mRNA expression in the 8 pair-wise comparisons.

### Kyoto encyclopedia of genes and genomes pathway and Sankey analyses of differentially expressed genes in the hippocampus of compulsive and non-compulsive rats

**Figure 3:**
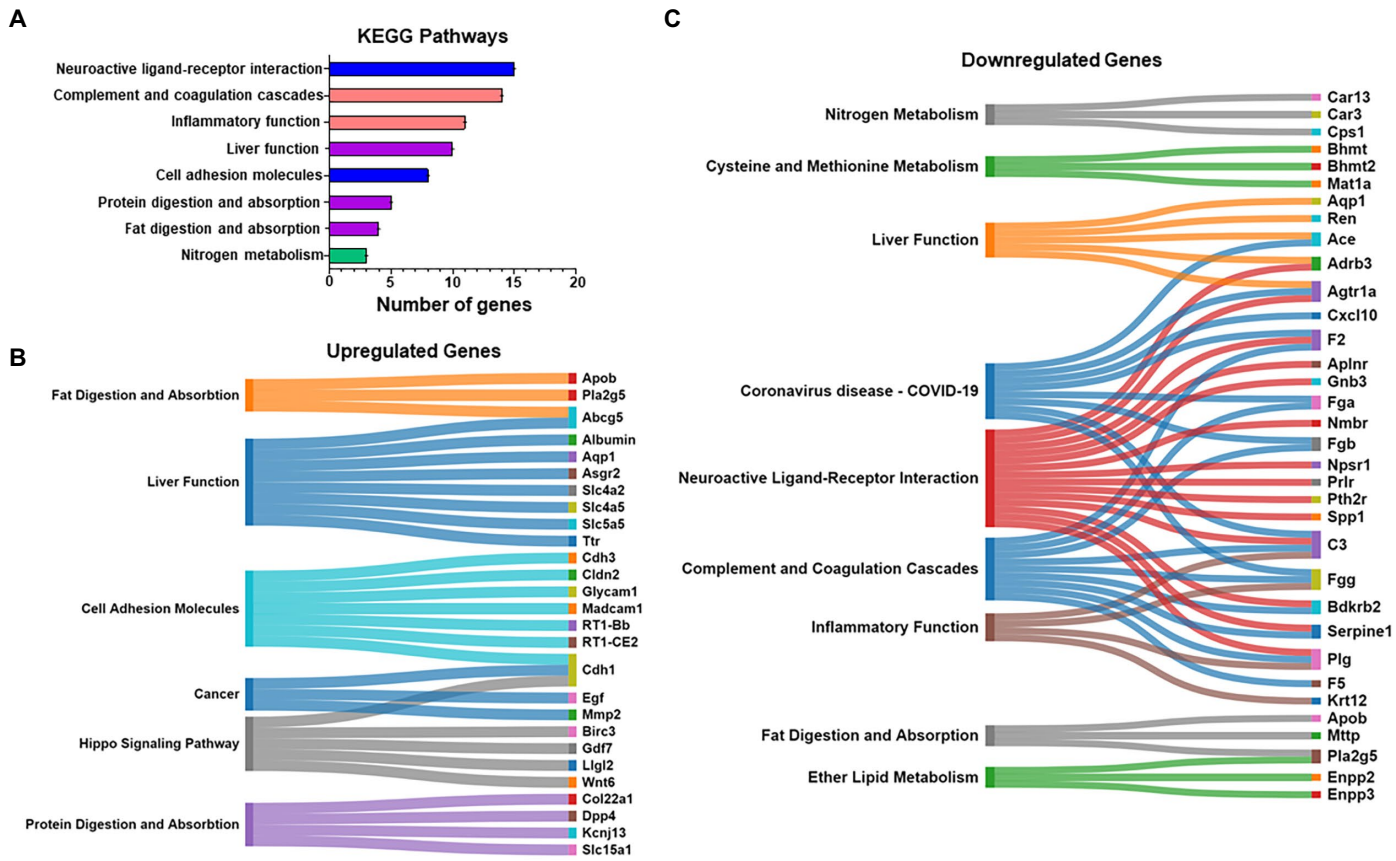
**(A)** Kyoto Encyclopedia of Genes and Genomes (KEGG) pathway analysis was also performed on the DEGs of all the eight pair-wise comparisons similar to the *Heatmap* in Figure 2L. A pathway prominently highlighted was cell adhesion molecules (CAMs). **(B,C)** The list of mapped genes included in the above KEGG pathways is represented as Sankey diagrams (Sankomatic.com) that display overlap of up- **(B)** and downregulated **(C)** genes involved in shared and unique KEGG pathways.

Kyoto encyclopedia of genes and genomes pathway analysis was also used to further identify relevant pathways that were differentially impacted in the compulsive and non-compulsive rats. Many DEGs participated in pathways related to neuronal ligand-receptor interactions, neuroinflammation, metabolism and absorption, and CAMs (Figure 3A).

We used Sankey diagrams to further show the interactions of up- (Figure 3B) and downregulated (Figure 3C) genes located in various KEGG pathways. The diagrams show that single genes that participate in multiple functions in the hippocampus are impacted by compulsive METH taking behaviors.

### Compulsive methamphetamine self-administration increased hippocampal expression of cell adhesion molecules

**Figure 4:**
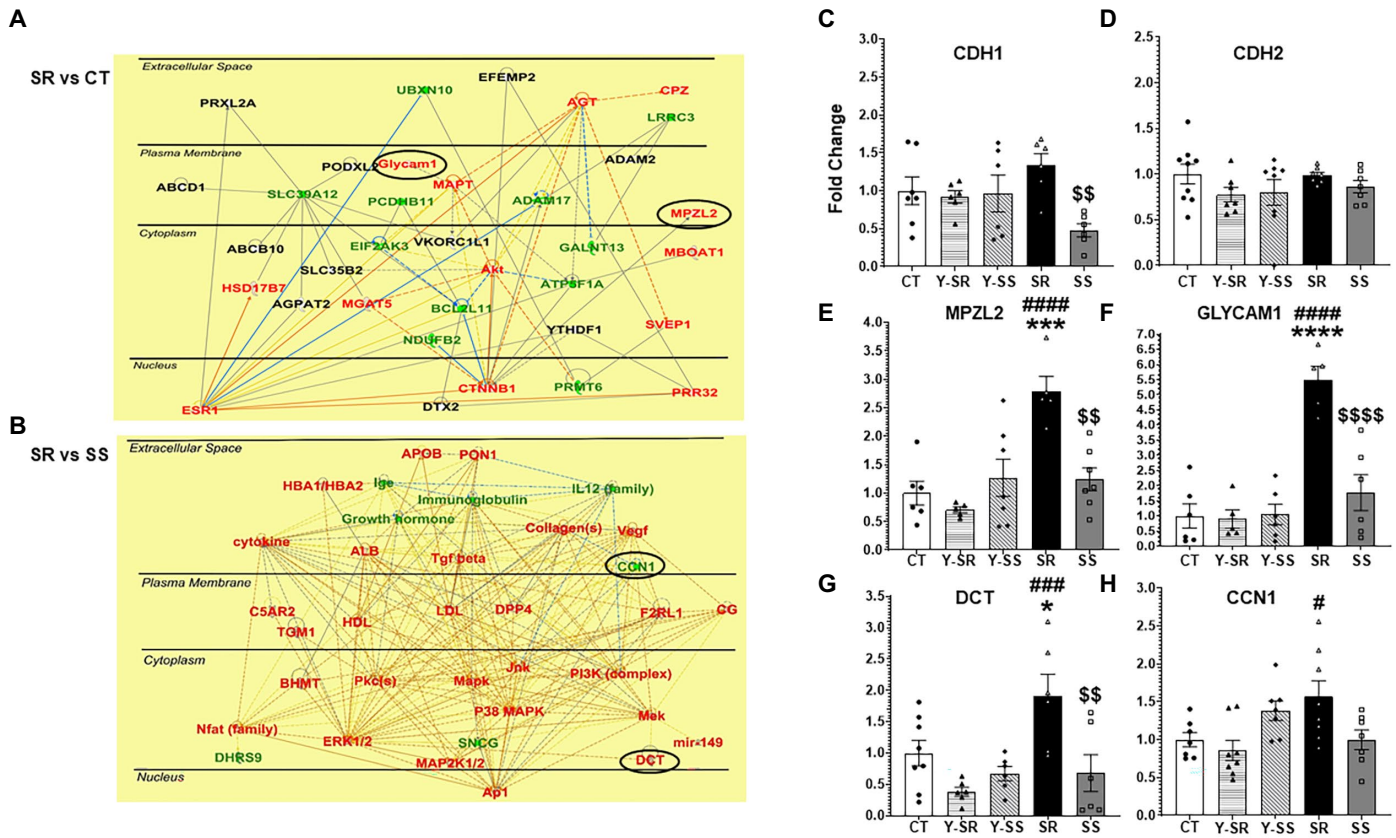
**(A)** Ingenuity Pathway Analysis (IPA, www.qiagen.com/ingenuity) shows the pathways that included CAMs like *Glycam1* and *Mpzl2* that exhibited higher mRNA expression in compulsive SR rats compared to CT rats. **(B)** IPA also illustrates other genes that *Dct* and *Ccn1* interact with. The red color represents upregulated transcripts whereas the green color represents downregulated transcripts. **(C–H)** We used qRT-PCR to validate the changes in some CAM-related transcripts identified by the sequencing experiments. The data in the bar graphs are shown as fold-changes in comparison to control. *Key to statistics:* *significant to CT, ^#^significant to paired YSR/YSS, ^$^significant between SR and SS; *p-values*: *p < 0.05, **p < 0.01, ***p < 0.001, ****p < 0.0001.

We also used Ingenuity Pathway Analysis (IPA) to identify biological networks affected by METH SA and foot-shocks (Figures 4A,B). Figure 4A showed upregulation of glycosylation dependent cell adhesion molecule (*Glycam1)* and myelin protein zero-like 2 (*Mpzl2*) in the comparison between the shock-resistant vs. -sensitive rats. Figure 4B showed that there was increased expression of L-dopachrome Tautomerase (*Dct*) in the resistant group in comparison to the sensitive rats.

To further validate the RNA sequencing results, we ran qRT-PCR using primers for some CAMs of interest. The PCR results showing fold-changes in mRNA levels are shown in Figures 4C–H. *Cdh1* (cadherin 1), a member of the cadherin family of CAMs was significantly decreased [*F*_(4,26)_ = 3.445; *p* = 0.0219] in the SS group compared to SR (Figure 4C). *Mpzl2* [*F*_(4,25)_ = 9.393; *p* < 0.0001], *Glycam1* [*F*_(4,23)_ = 18.62; p < 0.0001], and *Dct* [*F*_(4,27)_ = 6.290; *p* < 0.0010], all showed significant increases in the compulsive group in comparison to the other groups (Figures 4E–G).

## Discussion

The main findings of these experiments are: (*i*) all METH taking rats increased their active lever pressing for the drug during METH SA training. The application of contingent foot-shocks caused clear separation of rats into compulsive/SR and non-compulsive/SS rats; (*ii*) RNA sequencing identified 354 DEGs that met the criterion of ±1.7-fold changes at *p* < 0.05. We found further that (*iii*) the DEGs were involved in cell signaling, and binding and transport of ions, or were CAMs; (*iv*) IPA helped to highlight networks that included *Glycam1*, *Mpzl2*, and *Dct*. Below, we discuss the potential roles of hippocampal CAMs in mediating compulsive METH taking by rats in the presence of adverse consequences.

The observed differences in *Cdh1* mRNA expression between the compulsive and non-compulsive rats are reminiscent, in part, of the results of our previous microarray experiments that identified increased expression of cadherin 4 (*Cdh4*) mRNA in the dorsal striatum of compulsive rats (Krasnova et al., 2017). Together, these results suggest that members of that family of genes might be involved in promoting drug taking behaviors in the presence of foot-shock punishment. Cadherins are a family of transmembrane protein characterized by 5 CDH repeats that participate in calcium-regulated interactions (Troyanovsky, 2022). These proteins also play important roles in synaptic changes involved in learning and memory *via* their interactions with beta-catenin (Bozdagi et al., 2000; Huber et al., 2001). In addition, cadherins interact directly and indirectly with AMPA receptors (Saglietti et al., 2007; Silverman et al., 2007). Therefore, the significant decreases in Cdh1 mRNA expression in the non-compulsive rats suggest the possibility that continued compulsive behaviors observed even during punishment might be dependent, in part, to normal functioning of the cadherin-beta catenin adhesion complex.

We found, in addition, that *Mpzl2/Eva1* mRNA expression is increased in the compulsive rats in comparison to the other groups including the non-compulsive rats (see Figure 4E). *Mpzl2/Eva1* is highly expressed in the epithelial cells of choroid plexus and helps to regulate the permeability of the blood/cerebrospinal fluid barrier (Chatterjee et al., 2008). Although *Mpzl2/Eva1* expression has not been studied extensively in the peripheral or central nervous system, except for studies on its role in hearing loss (Bademci et al., 2018; Wesdorp et al., 2018), upregulation of *Mpzl2* expression has been reported in the substantia nigra (SN) in a MPTP mouse model of Parkinson’s disease (PD) using microarray analyses (Yeo et al., 2015). *Cdh1* expression was also increased by MPTP. When taken together with our present results in the compulsive rats, these results suggest that *Cdh1* and *Mpzl2/Eva1* might be co-regulated by substances that impact dopaminergic systems in the brain. It remains to be determined to what extent these two proteins might work in concert in the hippocampus to cause neuroadaptive changes that might affect compulsive drug taking.

The mRNA of another well-known CAM, *Glycam1*, was also upregulated in the hippocampus of compulsive rats, with this mRNA showing the highest fold change (5.5-fold; compare Figures 4E–G). Glycam1 is a sialomucin-like ligand for L-selectin (Imai et al., 1991; Lasky et al., 1992). It is involved in the regulation of inflammatory responses *via* its interaction with L-selectin (Ivetic et al., 2019). These observations support the notion that METH use disorder might involve the activation of neuroinflammatory cascades in the brain (Sekine et al., 2008; Agarwal et al., 2022), with METH-induced neuroinflammation being responsible, in part, for the learning and memory deficits reported in patients with MUD (Moszczynska and Callan, 2017; Paulus and Stewart, 2020). Neuroinflammation-induced changes in the basic processes that regulate synaptic plasticity (Cornell et al., 2022; Mancini et al., 2022) might also promote the perpetuation of substance use disorders.

The paper has some limitations. One of the limitations has to do with our use of only male rats in the present study. The accumulated evidence indicates that there exist sex-dependent differences in the behavioral and molecular consequences of METH self-administration (Daiwile et al., 2021, 2022a,b). Although many studies use male rodents to investigate molecular and biochemical mechanisms of substance use disorders (SUDs), the evidence is clear that there exist sex differences in the clinical courses and responses to therapies in those populations (McHugh et al., 2018, 2021). Sex differences in animal models of SUDs have also been reviewed (Quigley et al., 2021). Cadet (2021) has suggested that these differences might be dependent on both neurobiological and psychosocial/environmental determinants of SUDs. Sexual dimorphism was proposed to probably be secondary to potential interactions of licit and illicit substances with endogenous systems that might show baseline sex-based differences (Cadet, 2021). These ideas were supported by the work of Daiwile et al. (2022b) that had documented baseline differences in stress-related genes (2021) and in markers of dopaminergic systems in some brain regions. Daiwile et al. (2022a) have reviewed these issues in great length in the case of METH, and previous studies from our laboratory have investigated sex differences in some genes (Daiwile et al., 2021). Future studies have already been planned for us to measure regional and more global transcriptional responses in rats of both sexes.

Another limitation in the present study is that we measured gene expression in whole tissue collected from the hippocampus. This approach did not allow us to specify whether the changes were occurring in cells of specific neuronal or glial phenotypes or in specific hippocampal sub-regions. Given the importance of cellular diversity in various brain sub-regions of brain structures, we have been engaged in discussions to conduct these types of investigations in our future studies.

In conclusion, overexpression of cell adhesion genes in compulsive METH SA may influence hippocampus-based molecular changes affecting learning and memory processes in repeated METH users. These changes could include differences in synaptic plasticity and neurotransmission in the hippocampus and cause cognitive impairments (Huntley et al., 2002; Golsorkhdan et al., 2020). These cognitive changes might influence responses to treatment as well as relapses to drug seeking and taking behaviors. Future studies will focus on elucidating the manner by which CAMs might impact compulsive METH taking by measuring and manipulating their expression in specific cellular phenotypes of both sexes.

## Data availability statement

The datasets presented in this study can be found in online repositories. The names of the repository/repositories and accession number(s) can be found at: https://www.ncbi.nlm.nih.gov/geo/, GSE203268.

## Ethics statement

The animal study was reviewed and approved by National Institutes of Health (NIH) Guide for the Care and Use of Laboratory Animals (ISBN: 978-0-309-15401-7) and the study was approved by the National Institute of Drug Abuse Animal Care and Use Committee.

## Author contributions

JC and SJ conceived the study and designed the methodology. SJ and CM analyzed the differential expressed genes of the RNA sequencing data obtained from GeneWiz. CM performed the RT-PCR experiments and wrote the first draft of the article with contributions from SJ. SJ and BL performed the behavioral experiment. JC reviewed the final manuscript. All authors contributed to the article and approved the submitted version.

## Funding

This research was supported by funds of the Intramural Research Program of the DHHS/NIH/NIDA.

## Conflict of interest

The authors declare that the research was conducted without any commercial or financial relationships that could be construed as a potential conflict of interest.

## Publisher’s note

All claims expressed in this article are solely those of the authors and do not necessarily represent those of their affiliated organizations, or those of the publisher, the editors and the reviewers. Any product that may be evaluated in this article, or claim that may be made by its manufacturer, is not guaranteed or endorsed by the publisher.

## Footnotes

^1^ http://www.ncbi.nlm.nih.gov/geo/

^2^ http://www.interactivenn.net/
